# Genetic manipulation of rod-cone differences in mouse retina

**DOI:** 10.1371/journal.pone.0300584

**Published:** 2024-05-06

**Authors:** Ala Morshedian, Zhichun Jiang, Roxana A. Radu, Gordon L. Fain, Alapakkam P. Sampath

**Affiliations:** Department of Ophthalmology and Stein Eye Institute, David Geffen School of Medicine, University of California, Los Angeles, California, United States of America; University Zürich, SWITZERLAND

## Abstract

Though rod and cone photoreceptors use similar phototransduction mechanisms, previous model calculations have indicated that the most important differences in their light responses are likely to be differences in amplification of the G-protein cascade, different decay rates of phosphodiesterase (PDE) and pigment phosphorylation, and different rates of turnover of cGMP in darkness. To test this hypothesis, we constructed *TrUx;GapOx* rods by crossing mice with decreased transduction gain from decreased transducin expression, with mice displaying an increased rate of PDE decay from increased expression of GTPase-activating proteins (GAPs). These two manipulations brought the sensitivity of *TrUx;GapOx* rods to within a factor of 2 of WT cone sensitivity, after correcting for outer-segment dimensions. These alterations did not, however, change photoreceptor adaptation: rods continued to show increment saturation though at a higher background intensity. These experiments confirm model calculations that rod responses can mimic some (though not all) of the features of cone responses after only a few changes in the properties of transduction proteins.

## Introduction

Most vertebrate retinas detect light for vision with two kinds of photoreceptors: rods which are more sensitive but slower in response kinetics and principally responsible for perception in dim light, and cones which are less sensitive but kinetically faster and mediate bright-light and color vision [see 1]. In the developing retina, cones appear before rods [2], and cone visual pigments are evolutionarily more ancient than the rod visual pigment rhodopsin [3]. These observations make it likely that cones are the common ancestor of the two photoreceptor types and that rods evolved from cones. Rods and cones use similar phototransduction cascades but with many differences in protein structure and expression [see 4,5]. For some proteins such as the G protein transducin and the effector enzyme phosphodiesterase (PDE), rods and cones in most species express different isoforms [but see 6]. In contrast, the GTPase-accelerating proteins (GAPs), which are largely responsible for decay of activated PDE, have different levels of expression which can be 10-fold higher in cones than in rods [7,8]. These and other modifications are likely to be responsible for the differences in response kinetics and sensitivity of the two kinds of photoreceptors, with morphological differences such as the presence or absence of outer-segment disks making little or no contribution [9].

It remains unclear which of the molecular differences between rod and cone phototransduction are responsible for the differences in their physiological responses to light. In an attempt to identify those changes that are of greatest importance, Reingruber and colleagues [10] modeled dark-adapted rod and cone light responses from mouse photoreceptors and concluded that the most important differences were (A) transduction gain, reflecting differences in the amplification of the G-protein cascade; (B) variable rate of decay of the PDE and perhaps also of activated visual pigment; and (C) differences in the rate of turnover of the second messenger cGMP in darkness. To test this conclusion, we crossed two mouse lines, one of which had decreased transduction gain from decreased expression of transducin [11], and the other increased rate of PDE decay from increased expression of GAPs [12,13]. From these two alterations alone, the sensitivity of rods was brought to within a factor of 2 of the sensitivity of cones, after correcting for the difference in outer-segment dimensions. These changes, however, did not alter the properties of photoreceptor adaptation: mutant rods continued to show increment saturation though at a higher background light intensity.

## Results

We sought to understand how changes in phototransduction gain and the rate of PDE decay contribute to the difference between the response characteristics of rods and cones. Previous model calculations [10] suggest that making a simultaneous reduction in phototransduction gain while increasing the rate of PDE decay in rods may recapitulate much of the decrease in sensitivity observed in cones. To this purpose, we mated mice under-expressing rod transducin with reduced phototransduction gain to mice with increased expression of GAPs and accelerated PDE decay. The mouse lines used have been previously described and showed no evidence of retinal degeneration [11–13]. To confirm protein expression levels, we ran gels with a variety of antibodies for phototransduction proteins. As in previous studies [11–13], we found no significant differences among WT, *TrUx*, and *GapOx* animals in the level of expression of phototransduction enzymes such as the PDE alpha, beta, and gamma subunits; the transducin beta subunit; recoverin, GCAP-1 or GCAP-2; and guanylyl cyclase 1. That was also true for the *TrUx*/*GapOx* mice. There was no significant difference in Gnat1 expression between WT and *GapOx*, but levels in both *TrUx* and *TrUx*;*GapOx* were decreased to 0.16 ± 0.01 and 0.15 ± 0.06 of WT (S.D., n = 3 for both). Our estimates were similar though not as low as the 6% value given by Yue and colleagues [11]. For the GAP proteins R9AP and RGS9-1, we found no significant differences in expression between WT and *TrUx*, but both *GapOx* and *TrUx*/*GapOx* retinas expressed about 2 times as much GAP as WT, similar to the RGS9-ox line 1 of Krispel and colleagues [12]. We found that R9AP was expressed relative to WT at 1.6 ± 0.46 in *GapOx* and 1.9 ± 0.32 in *TrUx*/*GapOx*; Gβ5 at 1.4 ± 0.21 in *GapOx* and 1.6 ± 0.07 in *TrUx*/*GapOx;* and RGS9-1 at 2.3 ± 0.74 in *GapOx* and 3.0 ± 0.86 in *TrUx*/*GapOx* (n = 9, S.D. for all values).

### Light responses of TrUx/GapOx rods

**Fig 1:**
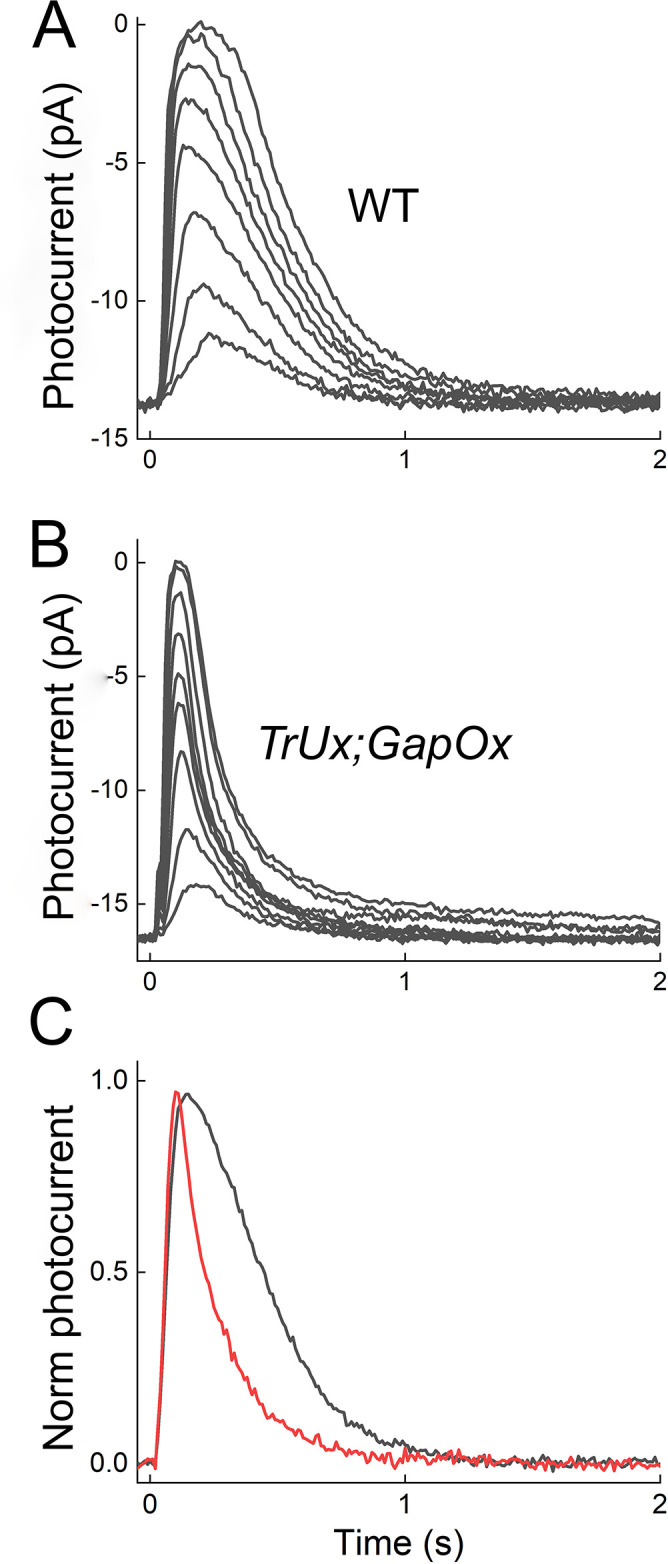
Suction-electrode recordings from WT and *TrUx;GapOx* rods. ***A*** and ***B***, Mean responses plotted as outer segment current for stimuli of 5.6, 11, 16, 35, 55, 73, 92, and 190 photons μm^2^ for WT rods (n = 12); and 93, 190, 380, 570, 760, 950, 2200, 3200 and 6500 photons μm^2^ for *TrUx;GapOx* rods (n = 10). Peak amplitudes averaged 14 ± 0.8 pA for WT and 17 ± 0.74 pA for *TrUx;GapOx*. ***C***, Comparison of half-saturating response waveforms in WT and *TrUx;GapOx* rods. Responses in WT (black, 16 photons μm^2^) and *TrUx;GapOx* (red, 380 photons μm^2^) closing approximately half of the channels open in darkness were normalized to their maximum amplitude and superimposed. An exponential decay function was used to calculate the time constant of recoveries τ_rec_, which were 250 ± 15 ms in WT and 130 ± 17 ms in *TrUx;GapOx*. The apparently more rapid rising phase of the mutant response is the result of the brighter light intensity used to elicit the half-saturating response.

**Fig 2:**
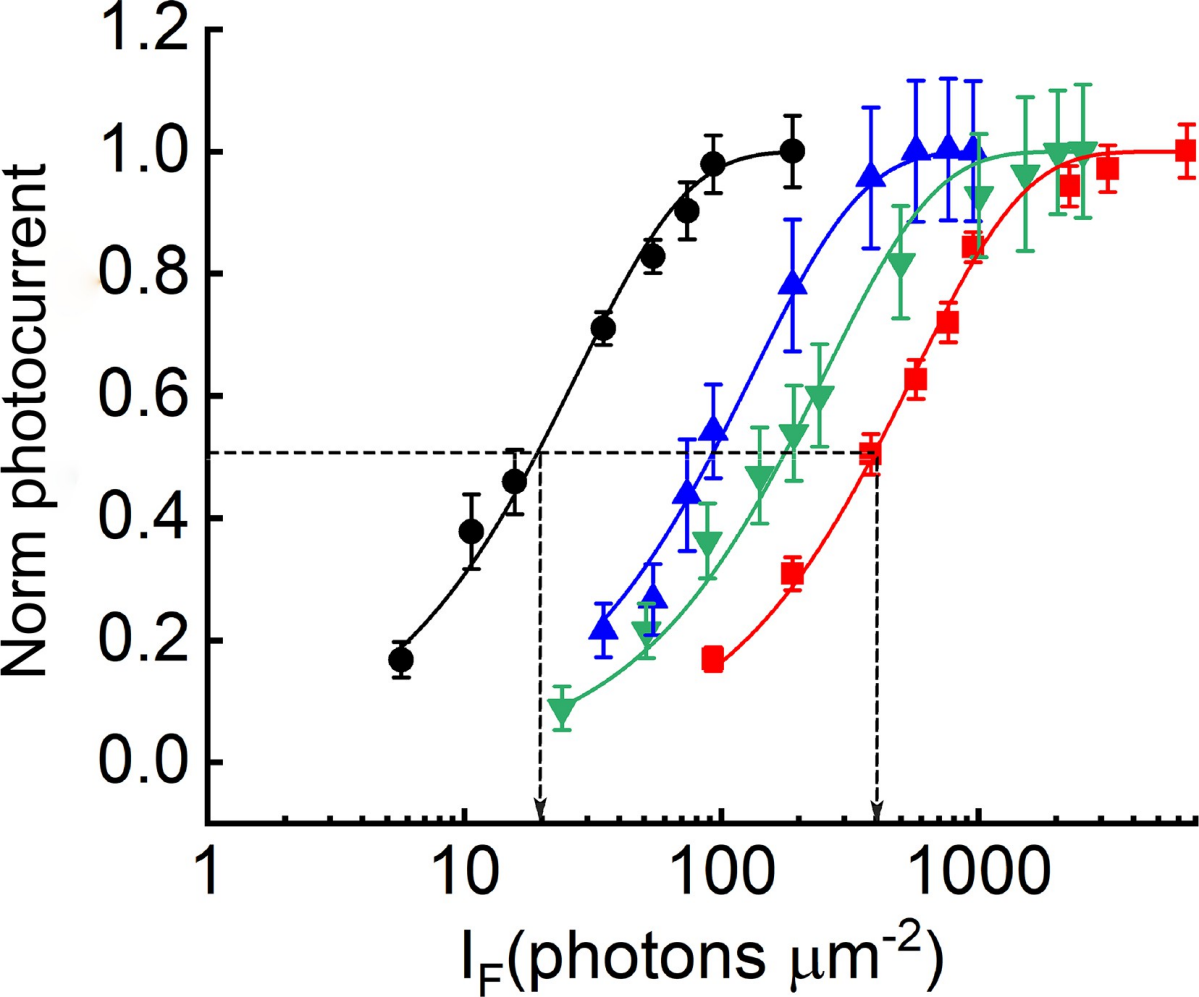
Normalized response-intensity curves. Data give means with SEMs for rods of WT (black circles), *TrUx* (blue, upward triangles), *GapOx* (green, downward triangles), and *TrUx;GapOx* (red, squares) Data have been fitted with an exponential saturation function (Eq 1), with values for *r*_*max*_ and *k* of 14 pA and 0.032 photons^-1^ μm^2^ (WT, n = 12), 20 pA and 0.0075 photons^-1^ μm^2^ (*TrUx*, n = 9), 11 pA and 0.0043 photons^-1^ μm^2^ (*GapOx*, n = 8) and 17 pA and 0.0017 photons^-1^ μm^2^ (*TrUx;GapOx*, n = 10), giving values of *I*_*1/2*_ of 22, 92, 160, and 410 photons^-1^ μm^2^.

In Fig 1, we compare the waveforms of the light responses of WT (*A*) and *TrUx*;*GapOx* (*B*) rods from the outer segment currents recorded with suction electrodes. There was little change in the peak amplitude of the dark-adapted responses: 14 ± 0.8 pA, (n = 12, S.E.) for WT; 17 ± 0.74 pA, (n = 10, S.E.) for *TrUx;GapOx*, (*p* < 0.001). There were however two salient differences between the light responses of the two lines. The sensitivity of the *TrUx*;*GapOx* rods was considerably lower than for WT photoreceptors. This difference can be appreciated by comparing the values of the stimuli for the flash series given in the legend to the figure but can be more easily seen from plots of response-intensity curves in Fig 2. These data show that both the *TrUx* rods (blue symbols) and the *GapOx* rods (green symbols) were less sensitive than WT rods [11,13], and that this difference was even greater for the *TrUx*;*GapOx* rods (red symbols).

Since the maximum amplitudes of the responses in the various lines were similar, we have compared the sensitivity of the rods by fitting normalized responses with an exponential saturation curve of the form [14],

$$r=r_{max}[1-\exp(-k\phi )]$$ (1)

where *r* is the peak amplitude of the response, *r*_*max*_ is the maximum value of *r* in bright light, *ϕ* is the number of photons per μm^2^ of the stimulus, and *k* is a constant with units of *ϕ* ^-1^ (photons^-1^ μm^2^). The value of *ϕ* required to produce a half-maximal response (*I*_*1/2*_) can be calculated from the constants *k* in Eq 1 and were 22 photons μm^-2^ for WT rods and 410 photons μm^-2^ for *TrUx*;*GapOx* rods (*p* < 0.00005). By this measure, the *TrUx*;*GapOx* rods were nearly 20-fold less sensitive than WT rods and within a factor of 2 of WT cones after correction for outer-segment dimensions (see Discussion).

The second important difference is that the light responses of *TrUx*;*GapOx* rods decayed more rapidly than WT because of the increased expression of the GAPs, as others have previously demonstrated [12,13]. In Fig 1*C*, we compare the normalized waveforms of half-saturating responses. The single-exponential time constants of decay (*τ*_*rec*_) averaged 250 ± 15 ms in WT and 130 ± 17 ms in *TrUx;GapOx* (*p* = 0.0002), with the value in *TrUx;GapOx* rods similar to that of 120 ± 13 ms observed in the RGS9-ox line 1 of Krispel and colleagues [12].

### Light adaptation of TrUx/GapOx rods: An initial test

The results in the first two figures show that reductions in gain and increases in GAP concentration in a rod can reproduce some of the physiological attributes of a cone. There is however another important difference between responses of the two kinds of photoreceptors. For rods, bright background light closes nearly all of the cyclic-nucleotide-gated (CNG) channels, with the result that the photoreceptors are initially completely saturated and unresponsive to superimposed flashes. Although rods in time can recover some of their responsiveness in bright light [15,16], response amplitude remains small. This behavior is in sharp contrast to that of cones, which escape saturation almost entirely and continue to give large responses even in the presence of light bleaching much of the photopigment [17,18].

**Fig 3:**
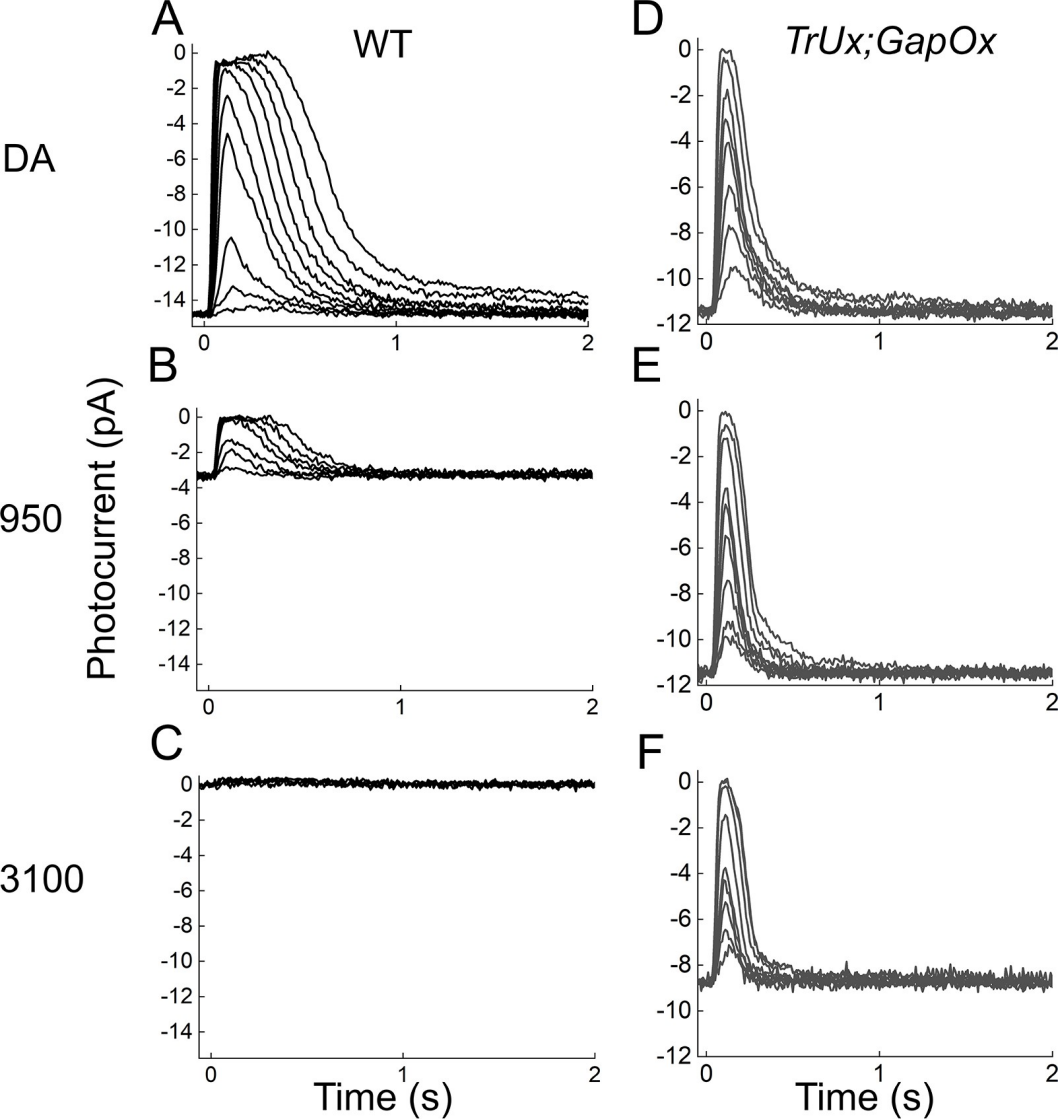
Comparison of the responses plotted as outer segment current of WT and *TrUx;GapOx* rods in the presence of background light. Responses were recorded in ***A—C*** from the same cell for WT and are representative of 8 experiments, and in ***D–F*** for *TrUx;GapOx* cell representative of 5 rods. ***A*** and ***D***, dark-adapted responses; ***B*** and ***E***, in presence of steady background light of 950 photons μm^-2^ s^-1^; and ***C*** and ***F***, in just saturating background of 3100 photons μm^-2^ s^-1^. Flash intensities (in photons μm^-2^) were: ***A*,** 2.4, 8, 21, 70, 120, 220, 400, 770, 1400, and 2600; ***B***, 71, 120, 220, 400, 770, 1400, 2600, 4700, and 8600; ***C***, 220, 770, 2600, 8600, 23000, and 75000; ***D***, 170, 350, 610, 970, 1300, 1700, 3400, and 7000; ***E***, 170, 350, 610, 970, 1300, 1700, 3400, 7000, and 11000; and ***F***, 350, 610, 970, 1300, 1700, 3400, 7000, and 11000.

In an initial attempt to determine whether *TrUx*;*GapOx* rods escape saturation like cones in background light, we recorded their responses to flashes superimposed onto three background illuminations and compared them to those of WT rods under the same conditions. These results are given in Fig 3. They show that the *TrUx*;*GapOx* rods are much less sensitive to backgrounds than WT rods and continue to give large responses when WT rods become almost entirely unresponsive. For short exposures to background light (less than 5 min), WT mouse rods can be saturated with a background illumination of about 3000 photons μm^-2^ at the λ_max_ of the photopigment (Fig 3*C*), as previous investigations have shown [for example 19,20]. At this background level, the *TrUx*;*GapOx* responses had a peak amplitude that remained a large fraction of the one recorded in the absence of a background (Fig 3*F*).

### Light adaptation of TrUx/GapOx rods: A more detailed study

**Fig 4:**
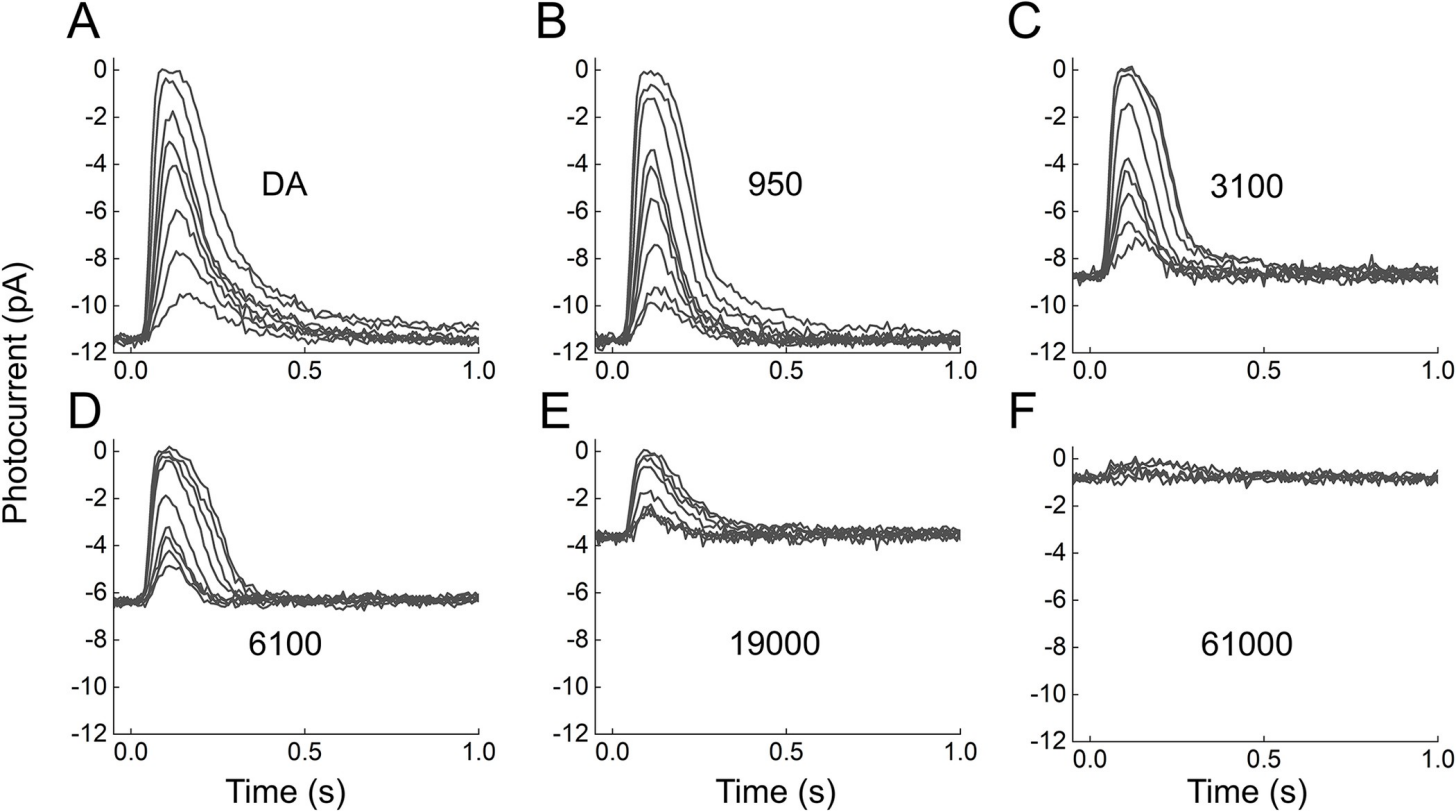
Light adaptation of *TrUx;GapOx* rods. Responses to 20-ms flashes plotted as outer segment current from rods dark-adapted or exposed to steady background light ***A–F***, means of five TrUx;GapOx rods in the dark (*A*) and in the following backgrounds (photons μm^−2^ s^−1^): 950 (*B*), 3100 (*C*), 6100 (*D*), 19000 (*E*), and 61000 (*F*). Values of *ϕ* were as follows (photons μm^−2^): ***A***, 170, 350, 610, 970, 1300, 1700, 3400, and 7000; ***B***, 170, 350, 610, 970, 1300, 1700, 3400, 7000, and 11000; ***C***, 350, 610, 970, 1300, 1700, 3400, 7000, and 11000; ***D***, 610, 970, 1300, 1700, 3400, 7000, 11000, 14000, and 18000; ***E***, 610, 970, 1300, 1700, 3400, 7000, 11000, 14000, and 18000; and ***F***,1700, 3400, 7000, 11000, 14000, and 18000.

**Fig 5:**
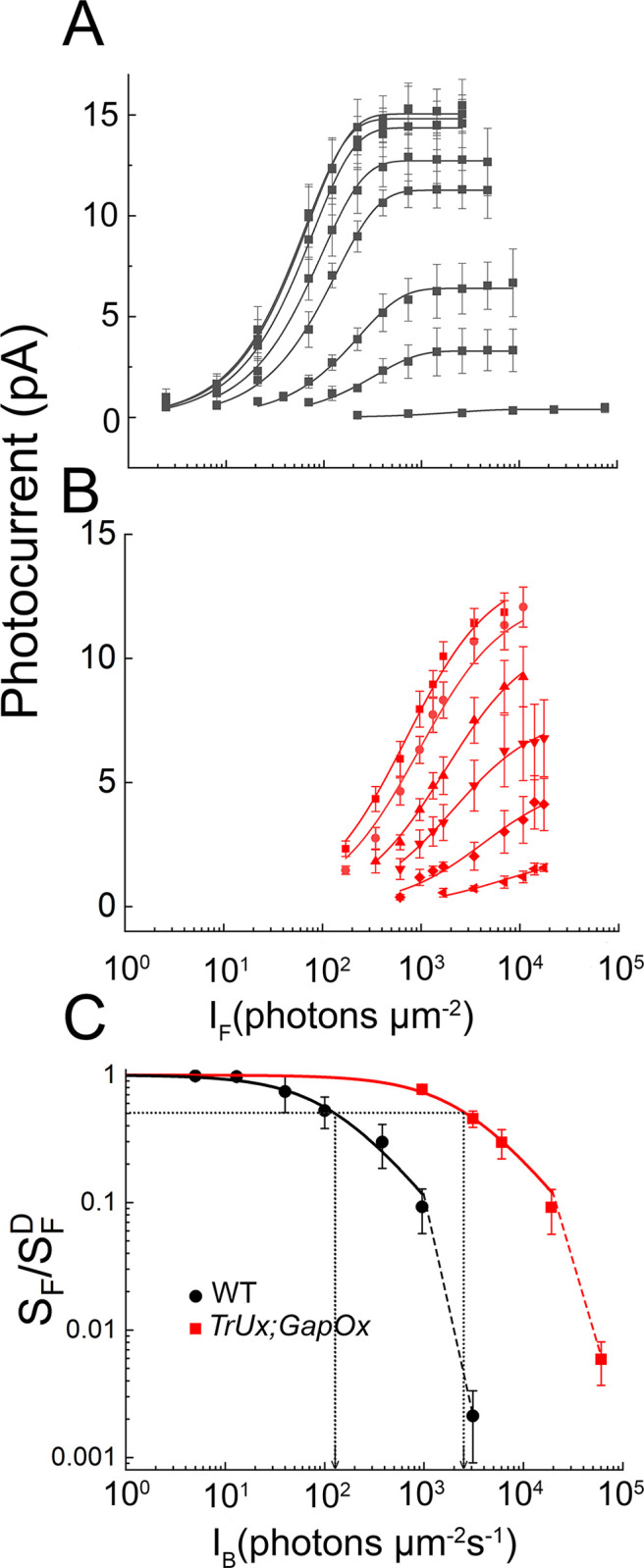
The effect of background light on WT and TrUx;GapOx rods: Response–intensity and Weber-Fechner curves. ***A***, Response-intensity curves for WT. Same cells as in Fig 3*A*–3*C*. Data points give mean response amplitude (with SE) as a function of *ϕ*. Data have been fitted with Eq (1) at the following backgrounds (in photons μm^−2^ s^−1^) with the following values of r_max_ and of *k*: dark-adapted (square), 15 and 0.016; 5 (circle), 15 and 0.015; 13 (upward triangle), 14.4 and 0.013; 40 (downward triangle), 13 and 0.011; 100 (diamond), 11 and 0.0075; 380 (leftward triangle), 6.4 and 0.0043; 950 (rightward triangle), 3.3 and 0.003; and 3100 (hexagon), 0.4 and 4.6 x 10^−4^. ***B***, Response-intensity curves for TrUx;GapOx. Same cells as in Fig 4*A*–4*F*. Mean response amplitude fitted with Eq (1) for the following backgrounds with the following values of r_max_ and of *k*: dark-adapted (square), 12 and 0.0012; 950 (circle), 12 and 7.9 x 10^-4^; 3100 (upward triangle), 9.1 and 5.5 x 10^−4^; 6100 (downward triangle), 6.6 and 4.3 x 10^−4^; 19000 (diamond), 4.1 and 2.4 x 10^−4^; and 61000 (leftward triangle), 1.6 and 1.6 x 10^−4^. ***C***, Weber-Fechner curves. Sensitivity relative to its value in darkness ($S_{F}/S_{F}^{D}$) plotted as a function of *I*_*B*_ for same rods as in *A* and *B*. Data have been fitted with Eq (2) with values of *I*_*0*_ of 130 photons μm^−2^ s^−1^ for WT rods (black curve) and I_0_ of 2600 photons μm^−2^ s^−1^ for *TrUx;GapOx* rods (red curve). A straight dashed line was drawn between the last two data points for both WT and *TrUx;GapOx* to show the departure of rods from the Weber–Fechner curve and approach to increment saturation.

It seemed to us possible that this difference in behavior may have been a reflection only of a difference in sensitivity of the WT and *TrUx*;*GapOx* rods to background illumination. We therefore explored the behavior of *TrUx*;*GapOx* rods over a larger range of background intensities. These results are illustrated in Figs 4 and 5. In Fig 4, we show a series of responses of *TrUx*;*GapOx* rods to increasing light intensities at 6 different background intensities. As the background intensity was increased to illuminations brighter than those used in Fig 3, responses became less sensitive and smaller. At the brightest intensity we tested, rod responses were barely detectable as the cells neared increment saturation. This behavior is easier to see in Fig 5*A* and 5*B*, where we have plotted the peak amplitude of the response as a function of the strength of the flash (*ϕ*) in photons μm^-2^ in several different background illuminations. The results for WT rods (Fig 5*A*) and for *TrUx*;*GapOx* rods (Fig 5*B*) are quite similar, but *TrUx*;*GapOx* rods are less sensitive to backgrounds by about 20 fold, the same amount as their sensitivity difference to flashes (Fig 2).

In Fig 5*C*, we have plotted the relative sensitivity of WT and *TrUx*;*GapOx* rods as a function of background intensity. The data have been fitted with the Weber-Fechner equation, namely,

$$\frac{S_{F}}{S_{F}^{D}}=\frac{I_{0}}{I_{0}+I_{B}}$$ (2)

where *S*_*F*_ is the sensitivity in the presence of the background light, $S_{F}^{D}$ is the dark-adapted sensitivity, *I*_*B*_ is the intensity of the background (in photons μm^-2^ s^-1^), and *I*_*0*_ is a constant called the “dark light”, equal to the background intensity required to reduce sensitivity by one-half. Both WT and *TrUx*;*GapOx* rods are well fit by this equation except at the brightest background, where sensitivity falls faster at the approach to increment saturation. The values of the constants *I*_*0*_, which are an indication of the sensitivity of the rods to the backgrounds, were 130 photons μm^−2^ s^−1^ for WT rods (black curve) and 2600 photons μm^−2^ s^−1^ for *TrUx;GapOx* rods (red curve) (*p* < 0.00002), which differ by a factor of about 20, nearly the same as the difference in the sensitivity of the rods to flashes.

### Dependence of response decay on background illumination

**Fig 6:**
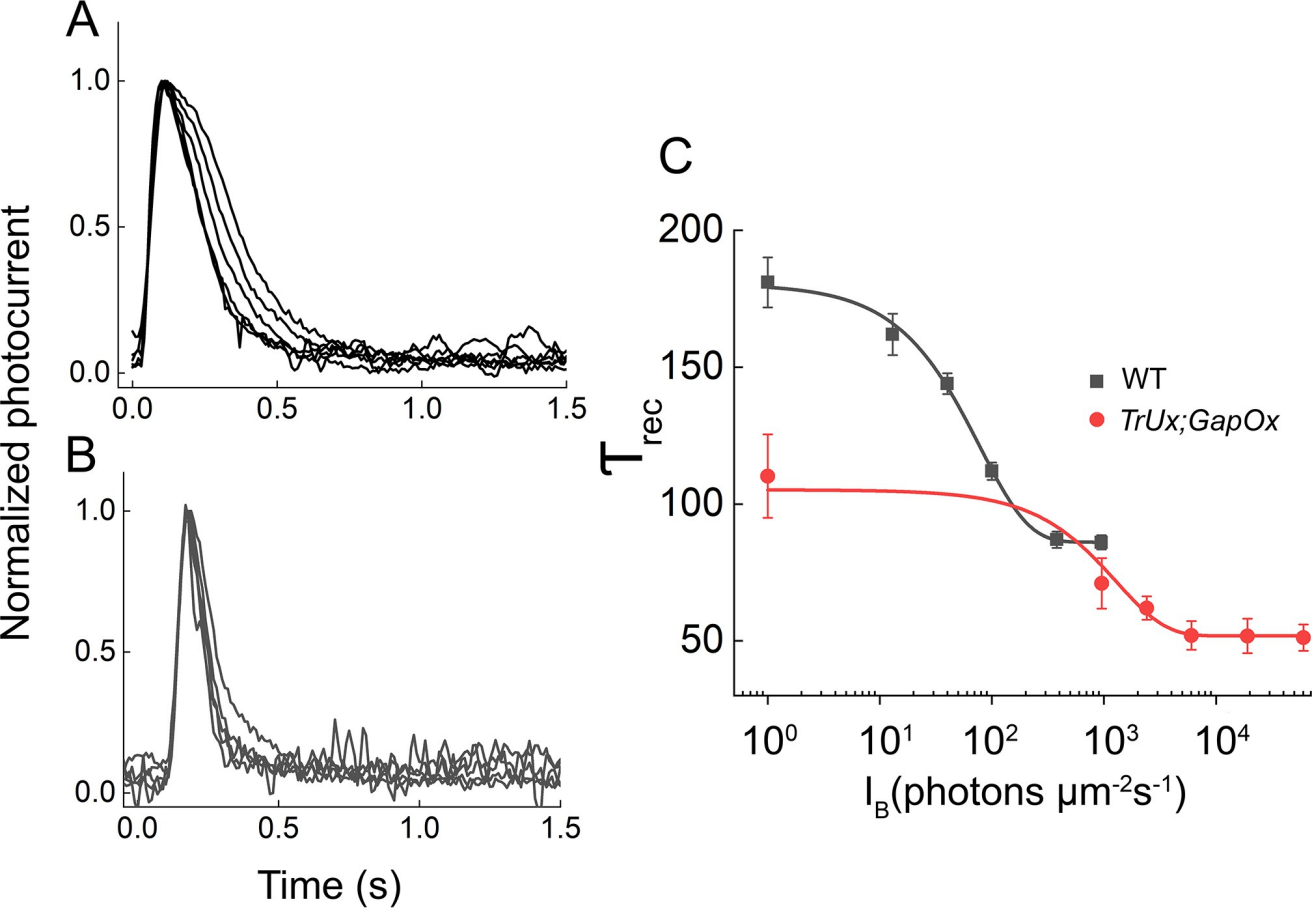
Time course of change in the kinetics of the response in the presence of background light. Average responses normalized to response peak as in Fig 1*C*. Responses are to flashes of light in the dark and in the presence of different intensities of background light for WT and *TrUx;GapOx* rods. ***A***, WT. Flash intensity for all responses was 220 photons μm^−2^ with background intensities of 0 (DA), 13, 40, 110, 380 and 950 photons μm^−2^ s^−1^. ***B***, *TrUx;GapOx* rods. Flash intensity for all responses was 1670 photons μm^−2^ with background intensities of 0, 950, 2400, 6100, 19,000, and 61,000 photons μm^−2^ s^−1^. ***C***, Recovery time constant (*τ*_*rec*_) for single-exponential decay fit to individual traces. The average values are plotted as a function of background intensity and fitted with a single-exponential decay function with decay constant of 78 ± 12 photons μm^−2^ s^−1^ (WT, black) or 1400 ± 150 photons μm^−2^ s^−1^ (*TrUx;GapOx*, red).

In addition to changing sensitivity, background light also alters the kinetics of the mouse rod flash response, speeding its decay [21]. In Fig 6*A*, we illustrate this effect for WT rods by plotting averaged and normalized responses to flashes of the same strength in the absence of a background and at several background intensities. As the background was increased, response decay was accelerated, and the single-exponential time constant of decay (*τ*_*rec*_) was shortened. In the brightest background, the value of *τ*_*rec*_ was decreased by about a factor of two. A similar phenomenon was also seen for *TrUx;GapOx* rods (Fig 6*B*), though responses were uniformly more rapid in decay. The value of *τ*_*rec*_ in the brightest background approached 50 ms, nearly that of a WT mouse cone [22].

In Fig 6*C*, we have plotted mean values of *τ*_*rec*_ for WT (black) and *TrUx;GapOx* rods (red) as functions of background illumination. The data have been separately fitted with single-exponential decay functions, whose best fitting constants were 78 ± 12 photons μm^−2^ s^−1^ for WT and 1400 ± 150 photons μm^−2^ s^−1^ for *TrUx;GapOx* (*p* < 0.00001). The ratio of these two values is about 18, again similar to the ratios for sensitivities to flashes (Fig 2) and background illumination (Fig 5*C*).

## Discussion

We have shown that from two changes in the expression of rod phototransduction proteins, namely a reduction in transducin and an increase in GAP protein expression, we were able to bring the flash sensitivity of a *TrUx/GapOx* rod close to that of a WT cone. The sensitivity of a rod to background light was also reduced, and by an equivalent amount. The adaptation of *TrUx/GapOx* rods to backgrounds nevertheless remained like that of WT rods, showing increment saturation though at a brighter background intensity, with no evidence of mechanisms that allow an escape from saturation like WT cones. These results demonstrate that differences in sensitivity and response kinetics between rods and cones could result from changes in the properties of only a very few transduction proteins, confirming previous model calculations [10]; but that differences in the response characteristics of the photoreceptors in bright light would require additional modifications to enable or prevent saturation.

### Changes in sensitivity

To change the sensitivity of the rod, we first employed *TrUx* rods to reduce the gain of phototransduction by reducing transducin expression to about 15% of that in WT rods. This alteration decreased the sensitivity of the rod by about 4 fold, in agreement with previous observations [11]. We further increased the expression of GAP proteins by about a factor of about 2, which by itself decreased the sensitivity of the rod by about a factor of 5. This change in sensitivity is greater than reported for the RGS9-ox line 1 of Krispel and colleagues [12], which also had an approximately two-fold greater expression of GAP proteins. The reason for this difference is unknown.

When we combined under-expression of transducin with over-expression of GAP proteins, we were able to bring the sensitivity of the resulting *TrUx/GapOx* rods to nearly 20-fold less than for a WT rod. Although the sensitivities of mouse rods and cones differ by about 70-fold [22], some of this difference is the result of the much larger outer segments and collecting areas of the rods. We can correct for these anatomical differences by multiplying the factor of 70 by the ratio of the cone-to-rod outer-segment volume (about 0.4, 23), which gives 28-fold; or by calculating the percent decrease in photocurrent per photon absorbed. Calculations of this kind give about 0.15% to 0.25% per pigment molecule beached for cones [23–25], and 5% for rods [26, see 27]. The resulting factor of between 20 and 30 is the difference in sensitivity produced by the phototransduction cascade alone and is within a factor of 2 of the difference between *TrUx/GapOx* rods and WT cones.

The aim of our study was to test model conclusions, that differences in sensitivity and kinetics between the two kinds of photoreceptors are largely the result of a small number of alterations in phototransduction proteins. Since with only two changes in protein expression, *TrUx/GapOx* rod responses can be made to have nearly the sensitivity of the dark-adapted light response of mouse cones, our experiments largely confirm the conclusions of previous model calculations. Although the sensitivity of *TrUx/GapOx* rods is similar to that of cones, we have not reproduced the cone phototransduction cascade in a rod since we made no effort to alter the rate of turnover of cGMP in darkness (see below).

### Changes in kinetics

The flash responses of *TrUx/GapOx* rods decayed more rapidly than WT rods but were not as rapid as WT cones, which have values of *τ*_*rec*_ in the range of 30 to 60 ms [22]. There are several possible reasons for this difference. Although GAP proteins in *TrUx/GapOx* rods were expressed at a level about two-fold greater than in WT rods, higher levels of GAP expression to 4-fold [12] or 6-fold [13] can produce progressively shorter values of *τ*_*rec*_. Although the amount of GAP in a mouse cone has not been determined, cones in other mammals can express GAPs at levels 10-fold higher than in rods [7,8]. WT cones probably also have a faster turnover of cGMP (*β*_*dark*_), which would contribute to the more rapid kinetics of cone responses [28]. Model calculations indicate that *β*_*dark*_ is likely to be about three-fold higher in a mouse cone than in a mouse rod [10], consistent with earlier experiments on amphibian cones [29]. We made no attempt to alter *β*_*dark*_ in our experiments.

### Adaptation to background light

Although *TrUx/GapOx* rods display some of the properties of cones, they continue to adapt to background light much like WT rods. As the experiments in Figs 3–5 show, exposure of *TrUx/GapOx* rods to steady illumination produced a progressive decrease in the maximum amplitude of the flash response and of sensitivity, whose dependences on background illumination were similar to those of WT rods provided the intensity of the background light was increased by about 20-fold. Moreover, *TrUx/GapOx* rods showed increment saturation just like WT rods, but at a 20-fold brighter background. In addition to changes in sensitivity, background light also produced an acceleration of the decay of the mouse rod flash response [21]. The results in Fig 6 show that a similar phenomenon also occurred in *TrUx/GapOx* rods, but again in backgrounds 20-fold brighter.

Our results show that changes in phototransduction proteins cannot by themselves reproduce the adaptational behavior of rods and cones. Rods saturate because the steady-state hydrolysis of cGMP by PDE is faster than synthesis of cGMP by guanylyl cyclase. Response saturation appears not to happen in cones exposed to bright light. This difference is unlikely to be the result of the cyclase or the Ca^2+^-dependent guanylyl-cyclase activating proteins (GCAPs), which are expressed to different degrees in rods and cones [30–33] but have similar enzymatic activities and Ca^2+^ dependence [34]; moreover, cones lacking GCAPs still escape saturate [22].

A more likely explanation for the difference is a more rapid shut-off of light-activated PDE in cones [see 35]. In dimmer background light, activated PDE is rapidly extinguished by an elevated concentration of GAPs, and cone pigment is turned off by phosphorylation by GRK1, which is known to be an essential step in cone response decay [36,37]. In brighter light, the GRK1 is apparently not abundant enough or fast enough [38] to extinguish the larger concentration of light-activated cone pigment [22,37], but cones nevertheless escape saturation because of the rapid decline of the pigment intermediates metaII [39] and metaIII [40] and the faster rate of cone pigment regeneration [41] aided by RGR opsin [42,43]. These mechanisms acting in concert allow the cone cGMP concentration to return nearly to its dark level in a very short time [44] and let cones continue to respond even in bleaching illumination [18].

### Evolution of photoreceptors

In the experiments we have described, we have attempted to alter the phototransduction cascade of a rod so that rod responses more nearly resemble those of cones. During evolution, however, it was the other way around: rods evolved from more ancient cones probably early in the Cambrian, facilitated by gene duplications [45,46] providing alternative isoforms of phototransduction genes [47]. Rods and cones in most vertebrates contain different genes for transducin and PDE, which are probably responsible for the greater gain of rod phototransduction. Substitution of cone transducin for rod transducin [48] and cone PDE for rod PDE [49] can produce changes in photoreceptor sensitivity which, together, could be as large as the one we have observed in the *TrUx* rods [but see also 50–52].

In addition, there must have been important changes in the evolution of photopigment structure, which for rhodopsin greatly slowed the time constants of decay of bleaching intermediates and increased the time required for pigment regeneration. These changes together with the decrease in GAP expression caused the rod response to saturate in brighter illuminations, with only modest recovery after prolonged light exposure [15,16].

These observations raise the question of what advantage rods acquired from saturation of the response and from the slower decay of pigment intermediates and regeneration. Rod saturation results in a large decrease in photoreceptor energy utilization [53,54], which is known to be an important driver during evolution [55]. We propose in addition that these changes may have been consequences of the greater stability of the rod pigment, which activates spontaneously in darkness at a rate many orders of magnitude lower than cone pigment [56–60]. This greater stability is essential to the high sensitivity of rod vision in dim light, which is limited by noise including that from spontaneous pigment activation [61]. We propose that the changes in rhodopsin structure responsible for its greater stability also produced slower decay of bleaching intermediates and pigment regeneration [62], which together with the lower GAP concentration were ultimately responsible for the different behavior of the rods and cones in bright light.

## Methods

### Animals and animal care

This study was conducted in accordance with the recommendations of the *Guide for the Care and Use of Laboratory Animals* of the National Institutes of Health, and the Association for Research in Vision and Ophthalmology Statement *Use of Animals in Ophthalmic and Vision Research*. Wild-type (WT) mice were Black-6 (C57BL/6) purchased from Jackson Labs (Bar Harbor, Maine, USA). Mice under-expressing rod transducin (Gnat1), which we term *TrUx*, are the same as the *Gnat1*^*Tg*^*;Gnat1*^*−/−*^ of Yue and colleagues [11]. In these animals, injection of a construct containing the *Gnat1* gene under the mouse opsin promoter together with deletion of the endogenous rod *Gnat1* gene resulted in under-expression of the Gnat1 protein to ~15% of WT rod Gnat1 (see Results). These mice were originally made and provided by the laboratory of Jeannie Chen at the Keck School of Medicine of the University of Southern California. Mice with over-expressed GAP complexes were made and provided by the laboratory of Ching-Kang (Jason) Chen at the University of Texas Health Science Center at San Antonio, by expressing a transgenic construct linked to the rhodopsin promoter to overexpress the anchoring protein R9AP, as previously described [12,13]. *TrUx;GapOx* mice were produced by mating the *TrUx* and *GapOx* lines. Verification of genotype was done conventionally with PCR as previously described [11,13]. Euthanasia was performed by cervical dislocation. Every effort was made to minimize pain and discomfort in mice used in this study.

### Immunoblotting

Retinas from each of the animals were homogenized in phosphate-buffered saline solution (PBS) with Halt protease inhibitor mixture (Life Technologies, Carlsbad, CA). Protein samples were treated with benzonase nuclease (Sigma-Aldrich, Burlington, Mad) at room temperature for 1 h and then rehomogenized with 1% sodium dodecyl sulfate (SDS) in PBS. Cellular debris was removed by centrifugation (20,000 x g, 2 min, 4°C), and protein concentration was determined with the Micro BCA Protein Assay Kit (Thermo Fisher Scientific, Waltham, MA). We then ran 10 μg of total protein from retinas of the different mouse lines on 4–12% or 12% SDS/PAGE gels (Novex, Thermo Fisher; Invitrogen, Thermo Fisher), except for antibodies to the GAP proteins R9AP, Gbeta5, RGS9-1. For these antibodies, we ran a series of protein concentrations between 1 and 10 μg of total protein to provide a quantitative assessment of protein expression. Membranes were blocked with Odyssey Blocking Buffer (LI-COR Biosciences, Lincoln, NE) followed by incubation at room temperature, and they were then probed with primary antibodies at a final dilution of 1 μg/ml. Antibodies used were as follows: PDEA (PA1-770, Thermo Fisher), PDEB (PA1-772, Thermo Fisher), PDEG (PA1-773,Thermo Fisher), Ros-GC1 (sc-376217, Santa Cruz Biotechnology, Dallas, TX), transducin alpha (Gnat1, sc-136143, Santa Cruz Biotechnology), GCAP1 (sc-136313, Santa Cruz Biotechnology), GCAP2 (sc-166056, Santa Cruz Biotechnology), recoverin (ab31928, Abcam, Cambridge, UK), and α-tubulin (T9026, Sigma-Aldrich). Antibodies for R9AP, Gβ5, and RGS9-1 were generously provided by Feng He and Theodore Wensel of the Baylor College of Medicine. Western blot analysis was performed with cognate IR dye-labeled secondary antibodies at a dilution of 1:50,000 and detected with an Odyssey CLx Infrared Imaging System (LI-COR).

### Suction-electrode recording

Methods for dissecting mouse retina and making suction-electrode recordings have been previously described [63]. Animals used for recording were younger than 6-months old and were selected approximately equally from either sex. Responses of single photoreceptor outer segments were recorded at 35–38°C with a current‐to‐voltage converter (Axopatch 200A, Molecular Devices, San Jose, CA), low‐pass filtered at 30 Hz with an eight‐pole Bessel filter (Kemo Limited Electronic Filters, Dartford, UK), and sampled at 100 Hz. Digitized data were recorded with Clampex, version 8.0 (Axon Instruments), and were analyzed with Origin Pro^®^ (OriginLab Inc., Northampton, MA). Curve fitting and plotting of data were also performed in Origin Pro^®^. Calculations of mean and variance were conducted either in Origin Pro^®^ or in Excel (Microsoft Corp., Redmond, WA, USA); values are given as the mean ± SEM unless otherwise stated. Statistical tests were performed with Origin Pro^®^ or MATLAB^®^ (see below). During recording, the photoreceptors were continuously perfused with Ames’ medium (Sigma Chemical, St Louis, MO, USA), containing an additional 1.9 g/l NaHCO_3_ and equilibrated with 95% O_2_ / 5% CO_2_. The recording electrodes were filled with Ames’ medium (Sigma Chemical, St Louis MO, USA) buffered with 10 mM HEPES to maintain pH = 7.4. Illumination was delivered with an OptoLED optical system (Cairn Research, Faversham, UK) and a 505-nm monochromatic LED nearly at the peak of spectral sensitivity of mouse rods [64]. The intensity of the light was controlled by the voltage output of the computer to the OptoLED optical system and was calibrated with a photodiode (OSI Optoelectronics, Hawthorne, CA).

### Statistical tests

Data are given as means plus or minus either the standard deviation (SD) or the standard error of the mean (SEM), as specified in the text and figure legends. Means were compared with the nonparametric Wilcoxon test in MATLAB^®^ or in Origin Pro^®^. Curve-fitting was done in Origin Pro^®^.
